# Disease dynamics over very different time-scales: foot-and-mouth disease and scrapie on the network of livestock movements in the UK

**DOI:** 10.1098/rsif.2007.1129

**Published:** 2007-08-14

**Authors:** Rowland R Kao, Darren M Green, Jethro Johnson, Istvan Z Kiss

**Affiliations:** Department of Zoology, University of Oxford Oxford OX1 3PS, UK

**Keywords:** livestock, foot-and-mouth disease, prion, social network, epidemiology

## Abstract

We analyse the relationship between the network of livestock movements in the UK and the dynamics of two diseases: foot-and-mouth disease (FMD), which has an incubation period of days, and scrapie, which incubates over years. For FMD, the time-scale of expected epidemics is similar to the time-scale of the evolution of the network. We argue that, under appropriate conditions, a static network analysis can be an appropriate tool for gaining insights into disease dynamics even when the relevant time-scales are similar, as with FMD. We show that a subclass of ‘linkage moves’ maintains the network structure, and so removing these links has a dramatic effect on the number of potentially infected farms, an effect corroborated by simulations. In contrast, because scrapie has a low probability of transmission per contact and a long incubation period, a static network representation is probably appropriate; however, the signature of the network in the pattern of transmission is likely to be faint. Scrapie-notifying farms were more likely to be associated with each other via trading at markets than were control farms; however, network community structure proves to be less representative of prevalence patterns than geographical region. These contradictory indicators emphasize that appropriate observation time frames and good discrimination among types of potentially infectious contacts are vital in order for network analysis to be a valuable epidemiological tool.

## 1. Introduction

The contact structure of a population can have consequences for disease transmission, such as when the variance in the number of potential contacts is high (Anderson & May 1992; Albert *et al.* 2000) or when transmission is localized but with occasional long-distance jumps (Watts & Strogatz 1998), and the network paradigm has become a popular one when modelling highly structured populations (Eubank *et al.* 2004; Meyers *et al.* 2005). While such studies have produced many interesting results, there are few datasets that include contact structure data that are both relevant to disease transmission in large populations and sufficiently well described to test the relevance of the network-based approach. One such dataset is the network of livestock movements in the UK, where the nodes of the network are agricultural premises and the links are the movements of batches of livestock between them.

The development of the livestock datasets in the UK has been largely motivated by a need to trace livestock movements for the purposes of disease surveillance and control. In particular, sheep, goat and pig movement data were first recorded extensively following the catastrophic foot-and-mouth disease (FMD) epidemic in 2001, where early dissemination of the disease was facilitated by the rapid, long-distance trading of sheep (Gibbens *et al.* 2001; Kao 2002). Records are maintained in the Animal Movements Licensing System (AMLS; http://www.defra.gov.uk/animalh/id-move/index.htm) and its Scottish equivalent (http://www.scotland.gov.uk/Topics/Agriculture/animal-welfare/Diseases/IDtraceability) administered by the Scottish Animal Movements Unit (SAMU), both recording the movements of large livestock (including sheep, pigs and goats) at the batch level. The FMD epidemic also saw the first extensive use of simulation and analytical models to inform disease control during an outbreak. These models used a combination of epidemiological and demographic data from the UK annual agricultural census (http://www.defra.gov.uk/esg/work_htm/publications/cs/farmstats_web/Census/introduction.htm) to inform disease control at the farm level (Ferguson *et al.* 2001; Keeling *et al.* 2001; Morris *et al.* 2001). Despite their limitations (Haydon *et al.* 2004), these data provide a picture of a uniquely well-described population, including spatial relationships between agricultural premises (most importantly for our purposes, farms and markets), information about the susceptibility and infectiousness of nodes (species mix and census population size) and the direction, timing (to nearest day) and weighting (number and species of livestock) of the livestock movements between them.

Prior studies have analysed the network of both cattle (Kao *et al.* 2006; Robinson & Christley 2006; Robinson *et al.* 2007) and sheep movements (Green *et al.* 2006; Kao *et al.* 2006; Kiss *et al.* 2006; Webb 2006). Here, we extend the analysis of the recorded sheep movement network, drawing particular attention to the difficulties and different approaches that are appropriate when examining two diseases (FMD and scrapie) that operate on very different time-scales, but interact on the same underlying population. We show that simple network analyses can give us insight into how a population is structured, but that this depends on the appropriateness of the recorded social network structure for the disease, both when seen through the filter of the transmission process and when considering the relative time-scales of disease and network dynamics.

Following the FMD epidemic, mandatory movement standstills after the inward movement of livestock onto farms were imposed to slow the progress of any incipient epidemic (http://www.defra.gov.uk/animalh/id-move/rules.htm). Thus, an interesting policy question is whether or not these restrictions have had an important effect on the probability of a large epidemic should FMD be reintroduced into the UK. The highly infectious FMD virus acts on a very short time scale with a farm-level infectious period of days or weeks; in this case, a small proportion of all sheep movements are shown to act as ‘bridging movements’ between well-connected groups of premises. We identify particular characteristics of these movements and demonstrate that a static network analysis is appropriate, despite the similar time-scales of network and disease dynamics. This is corroborated by simulations. In contrast, scrapie has a time-scale of years and lower infectiousness, and we show that, while there is some evidence of closer associations among affected premises via sheep movements, this signal is weak and subject to non-movement-related factors such as regional variation in farmer behaviour.

## 2. Critical network concepts for the livestock movement system

In most analyses of contact structure, the emphasis is placed on the properties of the social network of potentially infectious contacts (Watts & May 1992; Ghani *et al.* 1997; Liljeros *et al.* 2001; Meyers *et al.* 2003), i.e. which nodes *could* a node infect if it were infectious. For the tracing of potentially infectious contacts, the social network structure can be vital (Huerta & Tsimring 2002; Kiss *et al.* 2005, 2006). However, in the absence of control, or when control does not exploit social network structure, the analysis can be simplified by considering only the number of truly infectious contacts that *would* occur if a given node is infectious; we call this the ‘epidemiological’ or transmission network. The epidemiological network is created from the social network by ‘thinning’—links are discarded with a probability of $1-p\left(\tau_{i},\sigma_{j},w_{ij}\right)$, where $p\left(\tau_{i},\sigma_{j},w_{ij}\right)$ is the probability that, should the source node *i* be infected, destination node *j* would also become infected, given an inherent transmissibility *τ*_*i*_ of *i*, susceptibility *σ*_*j*_ of *j* and weighting of link *w*_*ij*_. The epidemiological network is generated from the social network stochastically, so a proper representation of average network properties could require multiple realizations (though in practice, single realizations of very large networks can give very good results). The epidemiological network also has directed links, even when the social network does not. Nevertheless, the result is a network of unweighted links with properties directly related to important epidemiological concepts. Three of the most important of these are the final epidemic size, the value of the basic reproduction number (*R*_0_) and population heterogeneity, here expressed in terms of communities of premises that are more likely to infect each other. These three characteristics are discussed below, in turn.

A component of a network is a group of linked nodes; in directed networks, a strongly connected component is a group of nodes mutually accessible to each other via a series of links, while a weakly connected component contains a strongly connected component (possibly of size zero) as well as nodes that are linked to that strong component in one direction or the other, but not both. The giant strongly connencted component (GSCC) is the largest strongly connected component, and, provided the network is ergodic (Kao *et al.* 2006), the GSCC of the epidemiological network is a direct estimate of the lower bound on the final epidemic size in the absence of intervention (e.g. the restriction of livestock movements that would occur following the confirmation of FMD). For our purposes, it is sufficient to say that, in an ergodic system (in this case, the network of farms linked by movements), any state of the system can be reached from any other via a Markov process (e.g. Reichl 1980); effectively, this means that the values and number of parameters that define the network structure are independent of the time in an epidemic at which the node is infected.

While the final epidemic size is an indicator of how large a problem might be caused by the introduction of a disease, *R*_0_ is an indicator of whether a problem will occur at all. It is generally defined as the number of secondary infections resulting from the introduction of a single infection into a wholly susceptible, homogeneously mixing population at equilibrium. If *R*_0_ is less than 1, a large epidemic is unlikely (Anderson & May 1992). However, this intuitively appealing definition is well known to be dependent on model assumptions. In a randomly mixed epidemiological network, *R*_0_ can be approximated by

(2.1) $$R_{0}=\frac{\langle l_{\text{in}}l_{\text{out}}\rangle }{\langle l_{\text{out}}\rangle },$$

where *l*_in_ and *l*_out_ are, respectively, the number of inward and outward ‘truly infectious’ links per node and the angled brackets represent the expectation value of the bracketed quantity (see appendix A for a more in-depth discussion).

Of course, populations are rarely well approximated as being random. While in the simplest epidemiological models all individuals interact equally with each other, population structure can often be usefully represented by metapopulation dynamics models where disease spreads among groups of populations, with strong homogeneous coupling within groups and weak coupling between groups. This group structure is related to the growth of the epidemiological network which increases with the probability of transmission; nodes that are more likely to infect each other will appear in the same components at low probabilities of transmission. If nodes are less likely to do so, then they will only ‘join up’ in the same component either when the probability of transmission is high or when they are linked by combinations of other nodes that are likely to infect each other. This property is related to network-based definitions of community structure, i.e. the existence of more closely linked nodes in subgroups of the population (e.g. Girvan & Newman 2002; Palla *et al.* 2005). However, most community structure algorithms are based on concepts that are appropriate for unweighted networks; for example, node ‘betweenness’ and ‘centrality’ are measures that both identify critical nodes for linking community subgroups (Newman & Girvan 2004) and do not consider the weighting of the links. A related community structure concept (Newman 2004) used in very large networks has been adapted and shown to be a useful concept for the livestock movement network (Green *et al.* 2006; Kao *et al.* 2006). In this algorithm, partitions are established that maximize the quantity

(2.2) $$Q^{\prime }=\sum_{i}e_{ii}-a_{i}^{\text{in}}a_{i}^{\text{out}},$$

where *e*_*ii*_ is the fraction of total links starting at a node in partition *i* and ending at a node in the same partition; $a_{i}^{\text{out}}$ is the fraction of links that begin in partition *i*; and $a_{i}^{\text{in}}$ is the fraction of links that end in partition *i*. The partition that maximizes equation (2.2) maximizes the difference in the amount of interaction that occurs within partitions compared with between partitions. This algorithm scales as *O*(*N*^2^ ln *N*) and is thus much better suited than most other existing algorithms for use in larger communities.

## 3. Network properties over short time-scales: FMD in the UK

### 3.1 Epidemiological background

FMD virus is a highly contagious pathogen that infects pigs and farmed ruminants, including cattle, sheep and goats. It can spread rapidly within herds and flocks, infecting large proportions of animals within days (see Haydon *et al.* (2004) for a review). The introduction of FMD into the UK in 2001 was exceptionally catastrophic, with control of the epidemic resulting in an estimated 8.5 million culled livestock and a combined direct and indirect cost of £4–6 billion (Anderson 2002).

The analysis of the rate of evolution of the FMD virus (Cottam *et al.* 2006) shows that introduction into the UK is likely to have occurred in early February; in the three weeks following this, the dissemination of FMD via sheep movements showed evidence of ‘scale-free’ properties (the importance of ‘superspreaders’ such as Longtown market) and ‘small-world’ properties (the occasional long-distance transport of sheep; see Kao 2002; Shirley & Rushton 2005; Kao *et al.* 2006; Ortiz-Pelaez *et al.* 2006).

### 3.2 Targeting high-risk movements

Recent analyses have considered the movements of livestock in the context of FMD since 2001 (Green *et al.* 2006; Kao *et al.* 2006; Kiss *et al.* 2006). Evaluation of *R*_0_ according to equation (2.1) shows the existence of percolation-like behaviour for *R*_0_ substantially above 1, associated with occasional, long-distance movement of sheep similar to those that were important in the 2001 epidemic. These conditions vary seasonally, with the network particularly vulnerable to invasion around early autumn. Comparing the growth of the GSCC during two different time frames (figure 1) shows that, while the GSCC growth is consistent with ergodicity within four-week periods, there are important structural changes in the network when comparing different time frames within the year.

The GSCC growth in autumn is markedly greater than at other times: farmers who purchase sheep from one market and immediately sell sheep at other markets are relatively few in number (of the order of 5% of recorded movements) and are critical for creating new foci of infection; we shall refer to these latter farm-to-market moves as ‘linkage moves’. The distribution of farms engaging in linkage moves is highly overdispersed, with tendencies towards scale-free properties, though over less than two orders of magnitude (figure 2*a*). Targeting linkage moves (Kao *et al.* 2006) and highly active farms (Kiss *et al.* 2006) have both been shown to be effective in targeting FMD control. Here we ask whether these properties can be exploited in concert: are highly active premises (i.e. highest total number of transactions, including both buying and selling) that link markets particularly important for transmission? Figure 2*b* shows the distribution of the number of linkage moves and number of premises engaging in linkage moves over 2005, and the expected variance-to-mean ratio (the overdispersion index *b*). The value of *b* increases as the distribution becomes increasingly overdispersed, from *b*=1, for a Poisson distribution, to b→∞, for a scale-free distribution. Increases in *b* are indicative of increased participation of highly active farms compared with the less active ones. As changes in the value of *b* show no long-term trends over 2005, this implies that, at any given time of year, highly active linkage farms are no more active than other farms performing linkage activities at any one time of year. Linkage movements from low- and high-activity farms also appear to have the same overall contribution to network structure. Figure 3 shows the growth of the GSCC as movements are added to a system initially without either farm-to-farm or linkage movements in the period from 3 to 31 October 2005, i.e. the four-week period of highest activity. As movements are added to the network, the GSCC size is measured as an indication of their contribution to any epidemic. Whether linkage movements are added in order either starting from the least active farms to the most or vice versa, the GSCC grows at the same rate, suggesting that the nature of the movements from both highly active and less active farms has the same characteristics. In contrast, adding farm-to-farm moves to the system causes the GSCC to grow more slowly, indicating that these are typically much less efficient at spreading an epidemic. The occasional large jumps in the GSCC size as links are added indicate the ‘absorption’ of smaller strong components into the GSCC.

The predictive power of this analysis was tested via ‘non-parametric’ simulations, to account for the effects of the timing and ordering of movements, factors that are lost in the static network analysis. Epidemiological parameters and rates of ‘local spread’ (i.e. other types of transmission not directly involving livestock movements) appropriate to the 2001 epidemic were used throughout, in this case considering only the sheep population (parametrization and methods described in detail by Green *et al.* (2006) and Kao *et al.* (2006)). The UK livestock population was seeded with five FMD-infected farms and then infection allowed to spread in a replay of the movements exactly as recorded. Considering 2000 iterations, epidemics starting on 3 October 2005±14 days and allowed to run for four weeks resulted in a mean of 7.3 premises infected in simulations without linkage movements and 7.9 premises with them. While this difference appears marginal, the number of linkage moves over this time frame is small (11 587 out of 499 361 movements over 2005 or just over 2% of the total) and so the relative effect is dramatic. Furthermore, epidemics of 60 or more infected premises are of similar extent to the initial dissemination in the 2001 epidemic and account for roughly 9% (174 out of 2000) of simulations. These are reduced by 27% if all linkage movements are removed (figure 4).

## 4. Network properties over long time-scales: scrapie

### 4.1 Epidemiological background

Scrapie is a neurodegenerative disease of sheep, goats and moufflon, which is generally believed to be the source of bovine spongiform encephalopathy (BSE) in cattle (http://www.defra.gov.uk/animalh/bse/publications/bseorigin.pdf). The discovery of the link between the fatal human disease variant Creutzfeldt–Jakob disease and BSE (Bruce *et al.* 1997; Hill *et al.* 1997), and concerns that BSE may have entered the sheep population and been masked by scrapie (Kao *et al.* 2002), have led to concerted efforts to eradicate scrapie, with control largely aimed at exploiting scrapie resistance in some sheep genotypes (Hoinville 1996). More recently, concerns that BSE could be maintained in putatively resistant genotypes (Houston *et al.* 2003; Kao *et al.* 2003), and the discoveries of BSE in a French goat (Eloit *et al.* 2005) and ‘atypical’ scrapie in sheep across Europe (Benestad *et al.* 2003; Le Dur *et al.* 2005), have presented new challenges to the development of policy, and motivate the development of targeted surveillance programmes that could better use our knowledge of the structure of the national flock to identify diseases that will appear with, at most, very low prevalence.

Scrapie has a long incubation period and is virtually undetectable until near the terminal point of the infection. Thus, buying activity has been identified as the most important risk factor for acquiring infection (McLean *et al.* 1999). Models of scrapie in sheep have considered the movement of sheep from premises to premises (Kao *et al.* 2001; Gubbins 2005), but without a detailed representation of the contact structure. We ask whether, even with the increased time-scale of scrapie transmission, the signature of the movement structure can still be seen in the patterns of scrapie-notifying farms, as is the case for a highly infectious, short time-scale disease such as FMD. The use of the sheep movement data suffers here from three problems. First, very few contacts will result in transmission, and thus the truly infectious contacts are likely to be only a small proportion of all movements. Second, the data themselves are not ideally suited for this use—movements of sheep occur for many purposes and may involve very short residence times. The high infectivity of the placenta (Race *et al.* 1998) and identification of lambing practices as a risk factor (McLean *et al.* 1999) would suggest that the residence time of sheep in infected flocks is important, at least as a surrogate variable for identifying sheep present at time of lambing. Third, most horizontal transmission is likely to occur on-farm, with no transmission events occurring at a market—this is in contrast to a highly infectious agent like FMD virus, which can be transmitted by short-duration events and fomite transmission. Thus, the inability to trace individual sheep passing through a market means that, while all farms buying sheep from a market selling infected animals must be considered as potentially infected, only a very few might be actually exposed.

Owing to these difficulties, a different approach to FMD is required. Here, we ask two network-related questions: are two scrapie-notifying farms more likely to be associated with each other by buying or selling at the same livestock market on the same day, and are scrapie-notifying farms more likely to belong to the same communities?

### 4.2 Farm interactions via markets

Because knowledge of scrapie may influence a farmer's trading behaviour, we consider only the combination of sheep movements in 2003 together with scrapie notifications in 2004 and 2005. In the UK (excluding the Shetlands, for which movement data were not available), there were 198 scrapie-notifying farms in the period from 2004 to the end of 2005 with usable records. These were paired with matching non-reporting farms in the same county. The geographical pairings were used to reduce differences in agricultural practices between farms; matching at finer geographical scales (e.g. parish) was not possible due to the limitations in the possible matches.

The number of interactions of case–control farms at markets (i.e. a buying farm moving sheep away from a market within 2 days of records of a selling farm bringing sheep to the same market) was calculated accounting for direction and time of movement. Within-pair comparisons using McNemar's test were used to determine whether farms of the same type are significantly more likely to associate with one another. These results are given in figure 5. For the 2004–2005 notification data, scrapie-reporting farms were more likely to sell sheep at markets where scrapie farms were buying (*p*=0.02). This partially reflects the fact that these farms are more active, making more movements on and off the premises in a year. However, if farm activity were the only factor, one would also expect to find control farms more likely to purchase from markets when scrapie farms were selling, but this was highly insignificant (*p*=0.68).

While the results are significant, they may not be important, as the number of farms involved in active trading is much lower in all cases than the number of farms that were inactive, with only 70 of the control farms and 76 of the reporting farms showing any associations at all. However, we must also consider that the time frame of exposure is probably much longer than the dataset (movements in 2003) we have available. Should farmers most often purchase sheep from ‘new’ trading partners, given that the number of possible interactions scales as *O*(*N*^2^), where *N* is the number of potentially interacting farms, the number of associations could increase linearly over the entire infectious period of a scrapie-affected farm, a period that could run into decades, especially since many within-flock epidemics could remain undetected (Hagenaars *et al.* 2003, 2006). Thus, were a dataset over a longer time frame available, the number of associations could increase markedly.

### 4.3 Distribution of scrapie among trading communities

The limitations of the data for determining direct associations suggest that a community-based approach may be more appropriate—i.e. are scrapie-affected farms more likely to belong to some communities of sheep-rearing farms rather than others? Five large communities based on all recorded movements in 2003 were resolved using equation (2.2). The resultant partitions are highly regionalized and presumably centred around local markets (figure 6*a*). Variations in the community-level prevalence of confirmed scrapie during the period 2004–2005 differed significantly from random in their distribution (*Χ*_4_^2^=62.015, *p*<0.001). For comparison, farm associations via geographical region were considered (figure 6*b*; table 1). If we assume that the distribution of disease is binomial, and use the prevalence of disease in ‘core’ elements (e.g. in table 1, in the community of 11 532 farms centred in northwest England, the core element is composed of the 5363 farms in the region defined as the northwest) as an estimate of the prevalence in ‘fringe’ elements (i.e. the 6169 farms in other regions), only in the Wales region are differences significant (*p*=0.02). However, differences are significant in all communities except North Wales, and, in this case, only 127 out of 3062 farms lie outside the core element. Since fringe elements of regions appear similar to their associated core element, but fringe elements of communities are dissimilar to their core element, it appears probable that region is a better indicator of the prevalence distribution of notified cases.

## 5. Discussion

Contact structure data with the detail found in the livestock movements database are invaluable for developing network models and for testing the validity of network concepts. Here, we show how the interaction of the social network and the epidemiological process is critical (see also Kao 2006; Trapman 2007). In this context, the epidemiological network representation is invaluable for determining the true impact of contact heterogeneity on disease transmission. While this representation is simple, complications will arise depending on other network properties. For example, where the in- and out-links are correlated (in extremis, where the links are bidirectional), the rate of link turnover relative to epidemiological properties becomes important. In our system, farmers typically will buy from one farm and sell to another, and so in- and out-links are poorly correlated. However, in the case of bidirectional links (i.e. complete correlation), link switching rates are critical (e.g. Watts & May 1992). While these limitations must be considered, so long as the infectiousness of a node does not depend on the level of its prior exposure to infection, the often complicated analyses required for weighted networks (Barrat *et al.* 2004) can be avoided.

Our two disease-network systems present very different challenges for analysis—the epidemiological network context is useful for FMD, but less so for scrapie where many of the recorded movements are likely to be unimportant for disease transmission. In the case of FMD, epidemics prior to identification of disease and the imposition of a national movement ban might be expected to last in the order of a month. As sheep movements vary seasonally, this is similar to the time-scale of the evolution of the network, and thus identifying when a static network analysis is useful is important. The ergodic hypothesis can help us here, indicating when changes in the network structure will change the potential for disease transmission. We note, however, that it remains only a supposition that the network is ergodic over this time frame—considering each year to be a replicate dataset, there are as yet only 3 years sufficiently well described to parametrize the underlying putative Markov process. Nevertheless, results thus far are consistent with the concept being useful, and structures identified by analysing the static network are shown to be valid in the dynamic simulations. In particular, analysing the effect of targeting a subclass of linkage moves that are largely responsible for connecting up the network, we show that removing these movements or links has a dramatic effect on the number of potentially infected farming premises. This is not proved to be a general result: more sophisticated analyses must be developed to characterize systems where the dynamics of the network itself become important. Nevertheless, removing linkage moves has a dramatic effect on the number and severity of very large epidemics and targeting them for surveillance or increased biosecurity may be a cost-efficient control policy. However, owing to the inherent variability in the system, and the expected short time-scale of any pre-movement ban epidemic, the signature of the network structure in a single realization of a simulated epidemic may not be noticeable, emphasizing the dangers of basing a policy on the outcome of only a few outbreaks, especially in cases where the underlying susceptible population is not well described or well known.

Scrapie presents a very different problem to FMD—the long infectious period of farms means that the static network picture is more likely to be appropriate; however, the signature of the network in disease transmission is likely to be faint, due to the low probability of transmission per potentially infectious contact. The prior evidence that sheep movements are important for scrapie transmission is strong, and this is corroborated by the evidence that scrapie farms are more likely to be associated with each other via buying and selling at markets. On the other hand, there is contradictory evidence showing that region is a better predictor of notification prevalence than trading community, thus the AMLS and SAMU data should be used with caution. Both analyses would benefit from a longer observation time frame and better discrimination among types of livestock movements. Interestingly, cross-comparison of the communities and regions shows that scrapie cases in the period 2004–2005 occurred most often in an area (South Wales) that traditionally has shown no evidence of high scrapie prevalence (Hoinville *et al.* 2000). These data could reflect a new outbreak confined within a regionally restricted trading group. However, there is also a risk that the non-random distribution of scrapie cases is an artefact reflecting regional control of scrapie monitoring or regionally varied responses to the Compulsory Scrapie Flock Scheme implemented in 2004 (http://www.defra.gov.uk/corporate/consult/tseregs-scrapiecomp/letter.htm). Thus, the strong correspondence between physical geography (which may be a surrogate for other non-epidemiological factors) and community structure must be accounted for when considering the use of trading communities as a marker of disease risk.

The difficulties associated with identifying network signature in disease transmission and control highlight the importance of good parametrizations of both epidemiological characteristics of diseases and the underlying demographic structure of the populations on which the epidemics transmit. More generally, extensive data are useful only if they are appropriate data and/or the appropriate questions are asked. As the demands placed on quantitative epidemiology become more extensive, more sophisticated analyses become increasingly important. Fortunately, the availability of improved datasets means that achieving these goals, while challenging, should be achievable in many important situations.

## Figures and Tables

**Figure 1:**
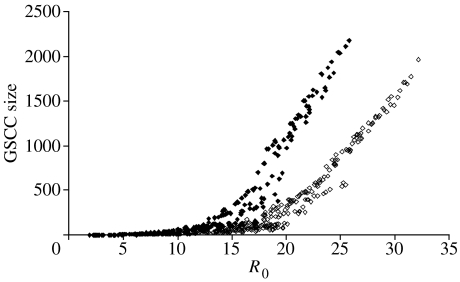
Growth of the GSCC with increasing *R*_0_ over two time frames (open symbols, four-week period starting 1 May 2003; closed symbols, four-week period starting 1 Nov 2003). Different values of *R*_0_ are obtained by a combination of increasing the probability of retaining a link from 0 to 1, and by increasing the infectious period of farms from zero to four weeks. Within each time frame, the growth of the GSCC is similar (consistent with ergodicity); between time frames, the growth of the GSCC is markedly dissimilar (not consistent). Adapted from Kao *et al.* (2006).

**Figure 2:**
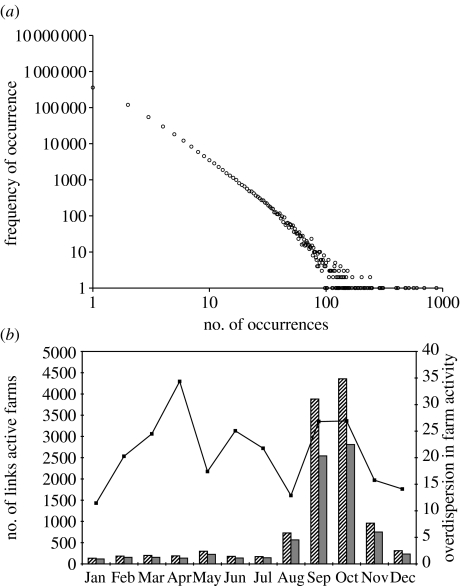
(*a*) The distribution of linkage movements per premises per week. The distribution is highly skewed, with approximately scale-free properties. (*b*) Linkage movement activity over 2005. Linkage moves are grouped in monthly periods. Graph shows the total number of linkage moves (diagonally hashed bars), the number of farms engaged in linkage activities (grey bars) and the variance-to-mean ratio *b* of the annual activity levels of farms engaging in linkage movements that month, weighted by the number of transactions in that month (black line). The value of *b* varies considerably but with no discernible annual pattern, suggesting that highly active farms are equally active in linkage moves over the entire year, but the number of active farms increases in the autumn.

**Figure 3:**
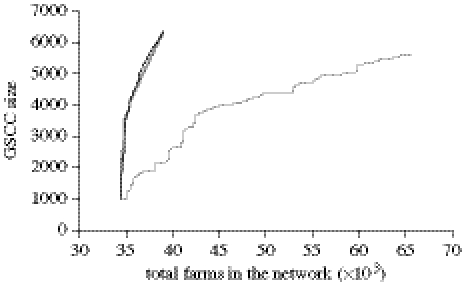
Growth of the GSCC as linkage and farm-to-farm moves are added. Linkage moves are added in the order of farmer activity, where activity is defined in terms of the total numbers of movements to or from a farm. Order is from least active to most active (black line) or most active to least active (hashed grey line), while farm-to-farm moves are added randomly (solid grey line). Order of linkage moves has no significant effect on the growth of the GSCC, suggesting that the characteristics of linkage moves from all farmers are similar. Farm-to-farm moves cause the GSCC to grow much more slowly than linkage moves, suggesting that they are a less efficient means of disease transmission.

**Figure 4:**
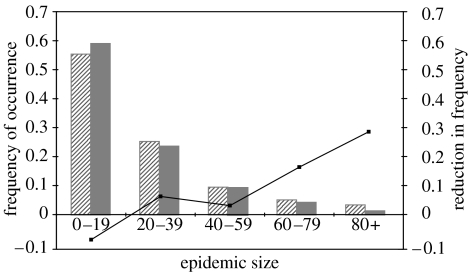
Distribution of FMD epidemic sizes predicted by non-parametric simulations run for four weeks. Data represent epidemics with all moves allowed (patterned bars), epidemics where linkage moves are removed (solid bars) and the proportionate differences between them (solid line).

**Figure 5:**
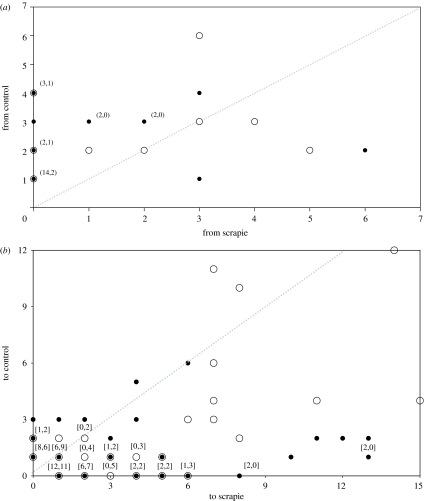
Distribution of number of times scrapie-notifying farms (open circles) and control farms (solid circles) when scrapie-notifying farms (*x*-axis) and control farms (*y*-axis) purchased (*a*) sheep at markets (buying behaviour) and (*b*) brought sheep to that market (selling behaviour) within the previous 48 hours. Scrapie farms were significantly more likely to buy when scrapie farms were selling (*p*=0.02). Numbers in brackets represent (number of control farms, number scrapie farms) where these are greater than one. Associations were found for 70 control farms and 76 scrapie farms. Not shown is the number of farms for which no associations were found (total of 152 pairs in the comparison). Also not shown are points representing single scrapie-notifying farms at (17,2), (18,18) and (20,12).

**Figure 6:**
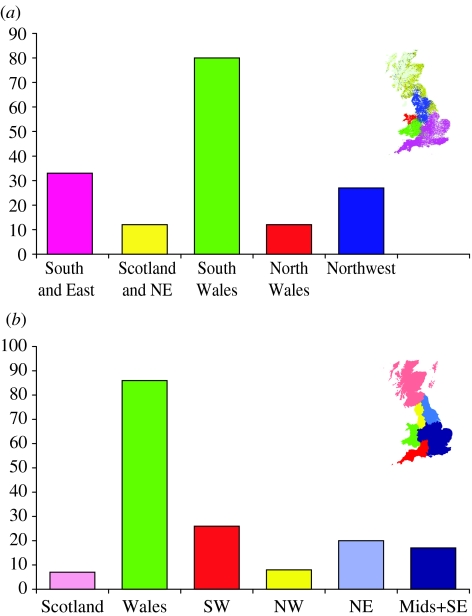
Distribution of scrapie cases over 2004–2005 in communities identified by (*a*) the ‘*Q*’ algorithm and (*b*) region. Data excludes cases in the Shetlands, for which no movement data were available. SW, southwest; NW, northwest; NE, northeast; Mids+SE, midlands and southeast.

**Table 1:** Distribution of scrapie cases over 2004–2005 in the five largest communities identified by (*a*) the ‘*Q*’ algorithm and (*b*) region. (Data from smaller communities (all fewer than 50 farms) are excluded, as are notifications and farms in the Shetlands, for which no movement data were available. Data are divided into core (e.g. for a given community, the single region with the largest number of farms) and fringe (e.g. for a given community, farms in all of the other listed regions). Shown are the number of farms and notifications in each category, and a *p*-value based on assuming a binomial distribution of cases, using prevalence in the core group as an estimate of the true prevalence in the fringe.)

	core farms	fringe farms	core notifications	fringe notifications	*p*-value
*region*					
Southeast and Midlands	5533	4062	8	9	0.06
Southwest	5803	341	23	3	0.11
Wales	7072	2959	74	12	0.00
Northwest	5363	133	8	0	0.82
Northeast	3808	2002	13	7	0.15
Scotland	5936	1166	6	1	0.36
*community*					
North Wales	2935	127	12	0	0.59
Northwest	5363	6169	8	27	0.00
South Wales	7072	3101	74	6	0.00
South and East	5803	5662	23	10	0.00
Scotland and Northeast	5936	2010	6	6	0.01

## Appendix A *R*_0_ in social networks

In general, one can define $R_{0}=\underset{N,n\to \infty }{\lim}\left(I_{n+1}/I_{n}\right)$, where *N* is the population size; *n* is the generation number; and *I*_*n*_ is the number of infected individuals in all classes in generation *n*. In a randomly mixed epidemiological network, *R*_0_ is the network percolation threshold (Cohen *et al.* 2002; Schwartz *et al.* 2002), loosely defined as the point at which the final epidemic size is expected to scale with the size of the population (discussed by Kao *et al.* (2006)). By randomly mixed, we mean that the probability of connection between nodes is directly proportional to the number of links to those nodes (with obvious extensions to directed networks). Following Kao (2006), the probability that a node of in-degree *κ*^in^ is connected to a node of out-degree *κ*^out^ is $P(\kappa^{\text{in}}|\kappa^{\text{out}})=\kappa^{\text{in}}p_{\text{out}}(\kappa^{\text{out}})/\langle \kappa^{\text{in}}\rangle$, where $p_{\text{out}}(\kappa )$ is the out-degree distribution of the network. For a random insertion of a single infected node into the population, and for a per link transmission probability *π*, the number of infected elements of an arbitrary in-degree *κ*^in^ for the first generation of transmission is

(A1) $$I_{\kappa^{\text{in}},1}=\pi \sum_{\kappa^{\text{out}}}P(\kappa^{\text{in}}|\kappa^{\text{out}})\kappa^{\text{out}}p_{\text{out}}(\kappa^{\text{out}})=\frac{\pi \kappa^{\text{in}}p_{\text{in}}(\kappa^{\text{in}})\sum_{\kappa^{\text{out}}}\kappa^{\text{out}}p_{\text{out}}(\kappa^{\text{out}})}{\langle \kappa^{\text{in}}\rangle }=\pi \kappa^{\text{in}}p_{\text{in}}(\kappa^{\text{in}}),$$

since the expectation value $\langle \kappa_{\text{in}}\rangle =\langle \kappa_{\text{out}}\rangle$. In the following generation:

(A2) $$I_{\kappa^{\text{in}},2}=\pi \sum_{\kappa^{\text{in}},\kappa^{\text{out}}}P(\kappa^{\text{in}}|\kappa^{\text{out}})\kappa^{\text{out}}I_{\kappa^{\text{in}},1}P^{\prime }(\kappa^{\text{out}}|\kappa^{\text{in}})=\pi^{2}\kappa^{\text{in}}p_{\text{in}}(\kappa^{\text{in}})\frac{\langle \kappa^{\text{out}}\kappa^{\text{in}}\rangle }{\langle \kappa^{\text{in}}\rangle },$$

where $P^{\prime }(\kappa^{\text{out}}|\kappa^{\text{in}})$ is the probability that a node with in-degree *κ*^in^ has out-degree *κ*^out^. It is easy to show using equations (A 1) and (A 2) and summing over all node degrees that *I*_2_*/I*_1_=*I*_*n*+1_*/I*_*n*_ for all subsequent successive generations *n* and *n*+1; therefore, $R_{0}=\pi \langle \kappa_{\text{in}}\kappa_{\text{out}}\rangle /\langle \kappa_{\text{out}}\rangle$. By extension, with weighted links and variable susceptibility of nodes,

(A3) $$R_{0}=\pi \frac{\langle \tau k_{\text{out}}\sigma k_{\text{in}}w\rangle }{\langle \tau k_{\text{out}}w\rangle },$$

where *τ* and *σ* are the weighting of the out- and in-links; *w* the weighting associated with each node; *k*_in_ is the number of inward links; and *k*_out_ is the number of outward links. This reduces to equation (2.1) in the epidemiological network, where *l*_in_ and *l*_out_ are, respectively, the number of inward and outward truly infectious links per node. Of course, most ‘real’ networks will have considerable structure and thus will not be randomly connected—in our example, farms preferentially buy and sell at particular markets, usually one to which there are in close proximity (Kao *et al.* 2006). The value of *ρ*(*M*), i.e. spectral radius of the epidemiological network contact matrix *M* (where an element *m*_*ij*_ is either 1 or 0, depending on whether there is an infectious contact between nodes *i* and *j*), is an alternative approximation for *R*_0_. While this explicitly accounts for the full contact structure of the network, the evaluation of extremely large, reasonably dense matrices (*O*(10^5^) nodes with some highly active nodes having hundreds of potentially infectious links) is difficult and time consuming, particularly when this must be repeated multiple times. However, comparisons between the two approximations for subsets of the sheep network with several thousand nodes show little difference in the two estimates (typically less than 5%; results not shown).

Should the rate of accrual of inward and onward links be fixed, equivalent values of *R*_0_ as determined by equation (2.1) or (A 3) can be derived by either increasing the probability that a potentially infectious link in the social network is infectious or increasing the average infectious period of nodes. Under these circumstances, differences in the GSCC size for the same *R*_0_ would imply that the network is not ergodic, and that the fundamental structure has changed in a meaningful way (Kao *et al.* 2006).
